# Incidence and complications of perioperative atrial fibrillation after non-cardiac surgery for malignancy

**DOI:** 10.1371/journal.pone.0216239

**Published:** 2019-05-07

**Authors:** Satoshi Higuchi, Yusuke Kabeya, Kenichi Matsushita, Nobuaki Arai, Keisei Tachibana, Ryota Tanaka, Riken Kawachi, Hidefumi Takei, Yutaka Suzuki, Masaharu Kogure, Yorihisa Imanishi, Kiyoshi Moriyama, Tomoko Yorozu, Koichiro Saito, Nobutsugu Abe, Masanori Sugiyama, Haruhiko Kondo, Hideaki Yoshino

**Affiliations:** 1 Division of Cardiology, Department of Internal Medicine II, Kyorin University School of Medicine, Tokyo, Japan; 2 Division of General Internal Medicine, Department of Internal Medicine, Tokai University, Kanagawa, Japan; 3 Department of Home Care Medicine, Saiyu Clinic, Saitama, Japan; 4 Department of General Thoracic Surgery, Kyorin University School of Medicine, Tokyo, Japan; 5 Department of General Thoracic Surgery, Nihon University School of Medicine, Tokyo, Japan; 6 Department of Surgery, Kyorin University School of Medicine, Tokyo, Japan; 7 Department of Otorhinolaryngology, Head and Neck Surgery, Kawasaki Municipal Kawasaki Hospital, Kanagawa, Japan; 8 Department of Anesthesiology, Kyorin University School of Medicine, Tokyo, Japan; 9 Department of Otolaryngology-Head and Neck Surgery, Kyorin University School of Medicine, Tokyo, Japan; Karolinska Institutet, SWEDEN

## Abstract

**Background:**

Perioperative atrial fibrillation (POAF) is one of the common arrhythmias in the setting of non-cardiac surgeries for malignancy. As POAF may cause subsequent adverse events, it is important to confirm its characteristics and risk factors.

**Materials and methods:**

The **pr**osp**e**ctive cohort stu**d**y of surve**i**llan**c**e for periopera**t**ive **a**trial **f**ibrillation **recurrence** (PREDICT AF RECURRENCE) is an ongoing prospective, single-center, observational study that aims to illustrate the clinical impact of POAF in major non-cardiac surgery for malignancy. Patients who planned to undergo non-cardiac surgery for definitive/suspected malignancy were registered. Those with a history of AF and atrial flutter were excluded. Any 30-day complications included acute myocardial infarction, congestive heart failure, bleeding, thrombosis, any infection, and acute kidney injury. The primary endpoint was an incidence of POAF.

**Results:**

The present study included 799 patients (age, 68 ± 11; male, 62%). Of these, 80 patients (10.0%) developed POAF. Notably, 66 patients (83%) had no symptoms. Any 30-day complications occurred in 180 patients (23%) (with POAF: 34 (43%); without POAF: 146 (20%); p < 0.001). POAF in 17 patients (50%) was preceded by the development of complications. No patient developed cardiogenic shock and/or acute heart failure. The association between 30-day complications and POAF development were analyzed using the multivariate adjusted model (odds ratio: 2.84; 95% confidence interval: 1.74–4.62; p < 0.001).

**Conclusion:**

Ten percent of patients who underwent non-cardiac surgery for malignancy developed POAF, which was strongly associated with perioperative complications. As a majority were asymptomatic, careful observation using electrocardiography monitoring is important to avoid oversights.

**Clinical trial registration:**

UMIN ID: UMIN000016146

## Introduction

Perioperative atrial fibrillation (POAF) in the setting of non-cardiac surgery is not uncommon; it was initially thought of as a temporal complication of surgery that does not affect the patient’s subsequent clinical course. However, recent studies have showed that POAF is associated with subsequent mortality and morbidity [1] and long-term anticoagulation might be beneficial in patients with POAF to prevent thromboembolism.[2] These results imply that AF can recur in patients with POAF who have undergone non-cardiac surgery. If this hypothesis is true, it is essential to detect POAF to prevent subsequent adverse events. Some non-cardiac surgeries are conducted for malignancies, which are associated with thrombosis [3] and/or bleeding.[4] The prediction of the incidence of POAF would be useful for short- and long-term management, such as anticoagulation therapy. As very few studies have reported on the clinical course and impact of POAF in the setting of non-cardiac surgery for malignancy, the present study aimed to reveal the incidence, characteristics, and predictors of POAF and its association with short-term mortality and morbidity.

## Materials and methods

### Study population

The **pr**osp**e**ctive cohort stu**d**y of surve**i**llan**c**e for periopera**t**ive **a**trial **f**ibrillation **recurrence** (PREDICT AF RECURRENCE) in major non-cardiac surgery for malignancy is an ongoing prospective, single-center, observational study that is designed to illustrate the clinical impact of POAF on mortality and morbidity. In this study, cardiologists collaborate with a surgical team during the perioperative period and investigate the frequency of AF recurrence after discharge in patients with malignancy. The study protocol has been reported previously.[5] In brief, patients who planned to undergo non-cardiac surgery for definitive/suspected malignancy were registered in the present study. The malignancies registered were as follows: head and neck (e.g., pharyngeal, laryngeal, tongue, mandibular, buccal mucosal, gingival, and glottic cancer), chest (e.g., esophageal and lung cancer, lung metastasis), or abdomen (gallbladder, pancreatic, and duodenal cancer, extra- and intrahepatic cholangiocarcinoma, carcinoma of the ampulla of Vater, hepatic cell carcinoma, and liver metastasis). Patients with gastric and colon cancers were not included because a majority of them were treated with less-invasive laparoscopic surgery in our institute and the incidence of POAF was expected to be low. Patients with persistent or chronic AF and atrial flutter were excluded from the cohort. Fourteen patients with a history of paroxysmal AF (PAF) were initially included in the cohort; however, they were excluded from the present analysis because one of the main purposes of the study was to reveal an incidence of POAF in those without a history of AF. The data and analyses of those with and without a history of PAF are provided in the supporting information tables. Patients who were treated from July 2014 to November 2017 were enrolled.

### Data collection

The baseline survey was conducted preoperatively to collect clinical information including age, sex, current medications, alcohol consumption, smoking status, past medical history of thoracotomy or cardiogenic stroke, and comorbid conditions such as heart failure, coronary artery disease, peripheral artery diseases, or chronic obstructive pulmonary disease. Blood examination and 12-lead electrocardiography (ECG) were performed simultaneously. Operation record comprised the stage of malignancy, type of surgery, extent of tumor resection, and pathological findings. The stage in each surgery was determined based on the Union of International Cancer Control (UICC) TNM classification seventh edition. All patients were observed with an ECG monitor device during and after surgery. The included postoperative complications were acute myocardial infarction (AMI) (the presence of a typical rise of troponin at least one value above the 99^th^ percentile upper reference limit and at least one of the following: symptoms of myocardial ischemia, new ischemic ECG change, development of pathological Q wave, new regional wall motion abnormality in a pattern consistent with an ischemic etiology, or identification of a coronary thrombus on angiography) [6]; congestive heart failure (any symptoms such as orthopnea, paroxysmal nocturnal dyspnea, or dyspnea on effort and B-type natriuretic peptide (BNP)≧100 pg/mL) [5]; bleeding [5]; thrombosis such as stroke, transient ischemic attack, ischemic bowel disease, and pulmonary embolism; any infections that needed antibiotics; acute kidney injury (AKI) (an increase in serum creatinine level of ≥0.3 mg/dL within 48 hours or ≥1.5 times from the baseline value [7]; and others (desaturation necessary for home oxygen therapy, ileus, pulmonary fistula, and pancreatic fistula). After discharge, the follow-up data regarding survival was collected via a face-to-face interview in an outpatient clinic, a telephone interview, or a medical record. The other follow-up data such as PAOF was collected via a medical record. All patients were scheduled to be observed with an ECG monitor device in the wards for a minimum of 24 h up to 30 days postoperatively; the monitoring started from induction of anesthesia and the duration was based on each surgeon’s discretion. AF was confirmed by ECG. Complains such as palpitation did not contribute to diagnosis of AF.

### Definition

POAF was defined as AF during and/or after surgery until the thirtieth postoperative day. AKI was defined as an increase in serum creatinine level of ≥0.3 mg/dL or ≥1.5 times from the baseline value, occurring within 48 hours post-surgery.[7] The CHADS2 score includes congestive heart failure, hypertension, age ≥75 years, diabetes mellitus, and prior stroke, transient ischemic attack (TIA), or thromboembolism (doubled). The score ranges from zero to six. The CHA2DS2-VASc score includes congestive heart failure, hypertension, age ≥75 years (doubled), diabetes mellitus, prior stroke, transient ischemic attack (TIA), or thromboembolism (doubled), vascular disease, age 65–74 years, and sex. The score ranges from zero to nine.[8] Major adverse cardiovascular events (MACE) was defined as a composite of nonfatal stroke, nonfatal myocardial infarction, congestive heart failure, and cardiac death within the 30th postoperative day.

### Endpoint

The primary endpoint was an incidence of POAF. The secondary endpoints were MACE.

### Ethical principles

This study protocol conforms to the ethical guidelines of the 1975 Declaration of Helsinki in line with the Ethical Guidelines for Epidemiological Research by the Japanese government. This study was approved by the ethics committee of Kyorin University Hospital (approval number: 488) and registered on University Medical Information Network (UMIN ID: UMIN000016146). Written informed consent was obtained from each patient before each surgery by the physicians.

### Statistical analysis

Numerical data are presented as mean ± standard deviation if the data followed a normal distribution. Otherwise, data are displayed as median and interquartile range (Q1–Q3) values. Categorical variables are expressed as absolute numbers or percentages. Continuous variables were analyzed using the unpaired Student *t* test and Mann–Whitney *U* test. The Fisher exact test or the χ^2^ test were used for categorical variables as appropriate. The association between the incidence of POAF and any comorbidities in those without a history of AF at the time of surgery was assessed using uni- and multivariate logistic regression analyses and expressed as odds ratio (OR), 95% confidence interval (CI), and *P* value. Variables with a *P*-value <0.10 were retained for the multivariate logistic regression analysis with least absolute shrinkage and selection operator (LASSO). Multivariate model included CHA2DS2-VASc score, chronic obstructive pulmonary disease, hemoglobin, and creatinine. Statistical significance was set at *P* < 0.05. All statistical analyses were carried out using Stata software, version 14 (StataCorp, College Station, TX).

## Results

### Patient characteristics

The present study included 799 patients (age, 68 ± 11; male, 62%). Of these, 80 patients (10.0% of all the patients) developed POAF (6 during operation; 74 post operation). AF with heart rate (HR) ≥100 beats per minute (bpm) was observed in 73 patients, AF with HR <100 bpm and ≥50 bpm in 5, and AF with HR <50 bpm in 2. Patient characteristics are shown in Table 1 (the data including patients with a history of PAF are provided in S1 Table). Those with POAF were older, had higher CHA2DS2-VASc score, lower hemoglobin levels, and slightly higher creatinine and BNP levels.

**Table 1:** Patient characteristics.

	All (n = 799)	POAF (n = 80)	No POAF (n = 719)
Age, years	68 ± 11	72 ± 7	67 ± 11
Male, n (%)	496 (62)	53 (66)	443 (62)
Body mass index, kg/m^2^	23 ± 3	23 ± 3	23 ± 3
Systolic blood pressure, mmHg	128 ± 17	128 ± 16	128 ± 17
Diastolic blood pressure, mmHg	77 ± 35	76 ± 13	77 ± 36
Heart rate, bpm	71 ± 12	73 ± 13	71 ± 12
Hypertension, n (%)	361 (45)	39 (49)	322 (45)
Diabetes mellitus, n (%)	150 (19)	18 (23)	132 (18)
Past history of heart failure, n (%)	5 (1)	0 (0)	5 (1)
Past history of cardiogenic stroke, n (%)	1 (0)	1 (1)	0 (0)
Coronary artery disease, n (%)	44 (6)	4 (5)	40 (6)
Chronic obstructive pulmonary disease, n (%)	210 (26)	28 (35)	182 (25)
CHADS2 score	0 (1–2)	0 (1–2)	0 (1–2)
CHA2DS2-VASc score	2 (1–3)	2 (2–3)	2 (1–3)
Brinkman index	360 (0–880)	550 (0–973)	340 (0–840)
Alcohol consumption, g/day	0 (0–20)	0 (0–18)	0 (0–20)
No malignancy, n (%)	46 (6)	2 (2)	44 (6)
Stage of Malignancy			
Stage 0, n (%)	79 (10)	3 (4)	76 (11)
Stage 1, n (%)	336 (42)	39 (49)	297 (41)
Stage 2, n (%)	142 (18)	19 (24)	123 (17)
Stage 3, n (%)	80 (10)	9 (11)	71 (10)
Stage 4, n (%)	161 (20)	10 (13)	151 (21)
Head and neck cancer, n (%)	35 (4)	1 (1)	34 (5)
Chest, n (%)	517 (65)	55 (69)	462 (64)
Lung cancer, n (%)	429 (54)	51 (64)	378 (53)
Metastatic lung cancer, n (%)	63 (8)	1 (1)	62 (9)
Esophagus cancer, n (%)	25 (3)	3 (4)	22 (3)
Abdomen, n (%)	250 (31)	24 (30)	226 (31)
Pancreatic cancer, n (%)	117 (15)	8 (10)	109 (15)
Carcinoma of the ampulla of Vater, n (%)	9 (1)	2 (2)	7 (1)
Duodenal cancer, n (%)	3 (0)	0 (0)	3 (0)
Intrahepatic cholangiocarcinoma, n (%)	4 (1)	1 (1)	3 (0)
Cholangiocarcinoma, n (%)	31 (4)	2 (3)	29 (4)
Gallbladder cancer, n (%)	13 (2)	2 (3)	11 (2)
Hepatic cell carcinoma, n (%)	33 (4)	3 (4)	30 (4)
Liver metastasis, n (%)	42 (5)	6 (8)	36 (5)
Laboratory data			
White blood cell count, 10^9^/L	6.1 ± 2.0	6.3 ± 2.2	6.0 ± 2.0
Hemoglobin, g/L	132 ± 15	129 ± 14	133 ± 15
Platelet count, 10^9^/L	222 ± 68	221 ± 76	222 ± 67
D-dimer, μg/L	300 (200–600)	400 (200–700)	300 (200–600)
C-reactive protein, mg/L	1.0 (0.0–2.0)	1.0 (0.0–2.0)	1.0 (0.0–2.0)
Sodium, mmol/L	140 ± 5	140 ± 2	140 ± 6
Potassium, mmol/L	4.3 ± 1.4	4.3 ± 0.4	4.4 ± 1.5
Chloride, mmol/L	105 ± 5	105 ± 3	105 ± 5
Calcium, mmol/L	2.35 ± 0.11	2.34 ± 0.11	2.35 ± 0.11
Magnesium, mmol/L	0.90 ± 0.14	0.89 ± 0.11	0.90 ± 0.14
Albumin, g/L	42 ± 5	41 ± 4	42 ± 5
Creatinine, μmol/L	63 (51–73)	65 (53–81)	62 (51–73)
B-type natriuretic peptide, ng/L	22 (12–38)	30 (17–44)	21 (12–37)
Thyroid stimulating hormone, mIU/L	1.7 (1.1–2.5)	1.7 (1.0–2.4)	1.7 (1.1–2.5)
Free T3, pmol/L	3.8 ± 0.7	3.8 ± 0.7	3.8 ± 0.7
Free T4, pmol/L	15.4 ± 3.7	15.3 ± 2.7	15.4 ± 3.8
Medication			
Beta blocker, n (%)	57 (7)	6 (8)	51 (7)
Beta stimulant, n (%)	70 (9)	11 (14)	59 (8)
Renin angiotensin system inhibitors, n (%)	200 (25)	24 (30)	176 (24)
Calcium channel blocker, n (%)	251 (31)	31 (39)	220 (31)
Prior chemotherapy, n (%)	94 (12)	5 (6)	89 (12)
Levothyroxine, n (%)	19 (2)	0 (0)	19 (3)
Anticholinergic agents, n (%)	55 (7)	9 (11)	46 (6)

POAF, perioperative atrial fibrillation

The Brinkman Index is defined as numbers of cigarette smoked a day times smoking years.

### New onset POAF and concomitant symptom

ECG monitors were used in the wards for a median (interquartile ranges) of 72 (48–120) hours in 799 patients. New-onset AF was recorded in 80 patients (10.0%). AF appeared 48 (27–80) hours after completion of surgery. First POAF was sustained for 2.3 (0.2–14.0) hours and cumulative time during hospitalization was 6.0 (0.5–16.5) hours. Notably, 66 (83%) had no symptoms such as palpitation or chest discomfort regardless of epidural anesthesia. All the symptomatic patients except for one had never had any symptom consistent with POAF until they developed the arrhythmia. A patient had developed similar palpitation prior to surgery; however, its duration was only a few seconds. Six patients each had one of the following heart diseases: sick sinus syndrome, dilated cardiomyopathy, hypertrophic obstructive cardiomyopathy, Wolff-Parkinson-White syndrome, vasospastic angina, and angina pectoris. No patients developed acute heart failure and shock state due to POAF. In ultrasound cardiograph, the left ventricular ejection fraction was 65 ± 6%. The long and short axis in the left atrium was 50 ± 8 mm and 39 ± 7 mm, respectively. E/A was 10 ± 0.4 and E/e’ was 9.4 ± 2.9. The estimated right ventricular systolic pressure was 21 ± 9 mmHg. No patient had any valvular diseases.

### Perioperative complications

Until the 30^th^ post-operative day, 180 patients (23%) developed any complications (with POAF: 34 (43%); without POAF: 146 (20%)). None of the patients died. The details of the complications are described in Table 2. A low incidence of MACE was noted. MACE occurred in 4 out of 799 patients (3 patients developed congestive heart failure; 1 patient developed AMI and embolic cerebral infarction). Non-cardiac complications such as any infection, bleeding, and AKI were observed more frequently in the patients with POAF. Among 34 patients with POAF who developed any of the complications, AF in 17 (50%) were preceded by the development of complications. MACE except for AMI occurred before POAF. A patient with cardiogenic cerebral infarction developed complications in the following order: stroke, POAF, and AMI. Notably, POAF did not precede stroke in this case.

**Table 2:** Details of complications.

	All (n = 799)	POAF (n = 80)	No POAF (n = 719)
Any complications, n (%)	180 (23)†	34 (43)‡	146 (20)††
Cardiovascular diseases			
Congestive heart failure, n (%)	2 (0)	1 (1)	1 (0)
Myocardial infarction, n (%)	1 (0)	1 (1)	0 (0)
Cerebral infarction, n (%)	1 (0)	1 (1)	0 (0)
Non-cardiovascular diseases			
Any infection, n (%)	110 (14)	23 (29)	87 (12)
Bleeding, n (%)	8 (1)	3 (3)	5 (1)
Acute kidney injury, n (%)	15 (2)	5 (6)	10 (1)
Others, n (%)	78 (10)	13 (16)	65 (9)
Any fistula, n (%)	59 (7)	9 (11)	50 (7)
Miscellaneous, n (%)	19 (2)	4 (5)	15 (2)

POAF, perioperative atrial fibrillation

Forty-two†, 8‡, and 34†† patients had multiple complications, respectively.

### Variables related to POAF

**Fig 1:**
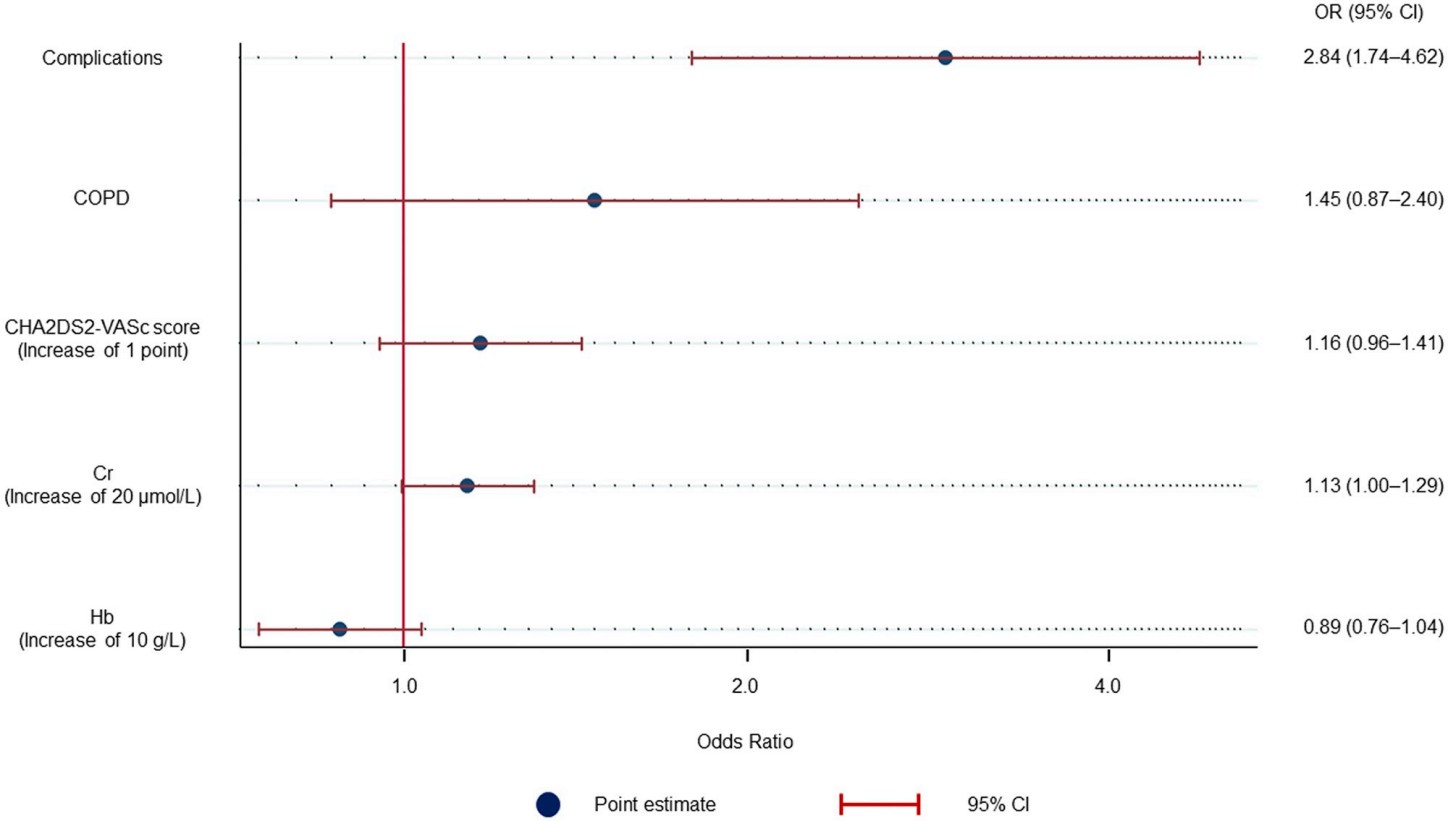
Multivariate logistic regression analysis for POAF development. Any 30-day complications were associated with POAF development. CI, confidence interval; COPD, chronic obstructive pulmonary disease; Cr, creatinine; Hb, hemoglobin; OR, odds ratio; POAF, perioperative atrial fibrillation.

Univariate logistic regression analysis indicated that any 30-day complications, CHADS2 score, CHA2DS2-VASc score, hemoglobin, and creatinine levels were related to the incidence of POAF (Table 3). Electrolytes were not predictors for the arrhythmia. Any 30-day complications were associated with the incidence of POAF (OR: 2.83; 95% CI: 1.75–4.57; p < 0.001). Multivariate logistic regression analysis demonstrated a similar result (OR: 2.84; 95% CI: 1.74–4.62; p < 0.001) (Fig 1). These results were similar if the patients with a history of AF were included (data included in the S2 Table).

**Table 3:** Univariate logistic regression analysis for an incidence of perioperative atrial fibrillation.

	OR (95% CI)	*P* value
Age (increase of 1 year)	1.05 (1.02–1.08)	< 0.001
Male	1.22 (0.75–1.99)	0.418
Chronic obstructive pulmonary disease	1.58 (0.97–2.58)	0.067
CHADS2 score (increase of 1 point)	1.30 (1.01–1.67)	0.042
CHA2DS2-VASc score (increase of 1 point)	1.22 (1.02–1.46)	0.029
Stage of malignancy (increase of 1 stage)	0.95 (0.79–1.13)	0.542
Primary / metastatic lung cancer	1.18 (0.73–1.91)	0.507
Hemoglobin (increase of 10 g/L)	0.85 (0.74–0.99)	0.037
Potassium (increase of 1 mmol/L)	0.79 (0.45–1.41)	0.430
Calcium (increase of 0.1 mmol/L)	0.88 (0.72–1.08)	0.230
Magnesium (increase of 0.1 mmol/L)	0.95 (0.77–1.18)	0.657
Albumin (increase of 10 g/L)	0.70 (0.43–1.13)	0.145
Creatinine (increase of 20 μmol/L)	1.15 (1.00–1.31)	0.042
B-type natriuretic peptide (increase of 100 ng/L)	1.44 (0.88–2.37)	0.145
Beta stimulant	1.78 (0.89–3.55)	0.101
Prior chemotherapy	0.47 (0.19–1.20)	0.114
Anticholinergic agents	1.85 (0.87–3.95)	0.109

### Anticoagulation for POAF patients

The CHA2DS2-VASc score was 2 (1–3) in males and 2 (2–3) in females. A score <1 in men and <2 in women was noted in 3 and 4 patients with POAF, respectively. After surgery, 69 patients (86%) were treated with anticoagulants according to study protocol. Of 11 patients who did not take the anticoagulants, 7 patients had no indication due to CHA2DS2-VASc score, 1 was due to hemodialysis, and 3 patients did not accept the treatment in spite of explanation regarding the risk of embolism. Anticoagulants were introduced in the patients 8 (5–13) days after surgery and 5 (2–10) days after AF development.

## Discussion

The present study provided some important findings. First, the most important finding was that a majority of patients with POAF had no symptoms and the median duration of POAF was only a few hours. This result suggests possible underestimation of the prevalence of POAF in previous studies[1, 9–11] because careful observation of ECG monitor would be the only means to detect asymptomatic AF. Second, a low incidence of MACE was noted even in the patients with POAF. Finally, POAF in non-cardiac surgery was strongly associated with peri-operative complications and did not yield severe results in the short term.

### Asymptomatic AF

If POAF was a temporal complication occurring only in the perioperative period, underestimation would not be serious because POAF in non-cardiac surgery might not cause severe problems. However, previous studies implied that POAF might recur in patients and lead to an embolic event.[1, 2] Therefore, documentation of POAF might be essential to prevent subsequent embolic events even in the asymptomatic patients. Whether POAF is a temporal arrhythmia and what is its recurrence rate will be revealed in our cohort study in future.

### POAF and cardiac complications

The present cohort included few patients with severe heart diseases. POAF did not induce any adverse cardiac disease such as cardiogenic shock and congestive heart failure. The finding was compatible with that in patients with AF and normal cardiac function. In the setting of non-cardiac surgery, AF might not significantly affect hemodynamics.

### Speculation of causal relationship between POAF and comorbidities

AF is known to be related to worse outcome in heart diseases such as congestive heart failure and coronary artery disease.[12–17] Although a few studies reported potential efficacy of rhythm control by catheter ablation on prognosis in patients with systolic dysfunction,[18] an advantage of rhythm control to prognosis over rate control has not been confirmed.[19, 20] It is difficult to determine the chicken-and-egg relationship between AF and heart failure, because each can cause the other. Some patients in the present study developed cardiovascular events such as congestive heart failure, myocardial infarction, and stroke. Our cohort could speculate their causal relationship with chronological evaluation. All the cardiovascular events except for AMI appeared prior to the incidence of POAF. AF may be a marker for severity of underlying disease in specific patients. Concerning non-cardiac complications, it would not be difficult to comprehend its relationship to AF. AF does not generally induce any infection or bleeding; these constituted majority of the complications seen in the present study. POAF would be the consequence of both cardiac and non-cardiac complications in this population. However, it was noted that half of the POAF patients did not develop complications prior to the first instance of AF. Causes of POAF in such subgroups may not be due to any complications, but to sympathicotonia derived from surgery.[21]

### Limitations

First, this was a single-center study and included limited subsets of non-cardiac surgery and there were a small number of patients with impaired cardiac function. The results acquired from the study may not be adapted for the other subsets. Second, although LASSO could minimize confounding, unknown confounding factors might have affected the results. Third, the type of cancer surgery was heterogenous. As the present study included many high-risk surgeries such as pancreaticoduodenal resection, the results may not be adaptable for less invasive surgeries such as mastectomy. Finally, the incidence of POAF might be underestimated because most patients with POAF were asymptomatic and ECG monitoring was not conducted for 30 days in most patients. However, the present study would provide some important information regarding the incidence of POAF and its relationship with adverse events in the setting of non-cardiac surgery for malignancy.

## Conclusions

The incidence of POAF was 10% in the setting of non-cardiac surgery for malignancy. Most patients with POAF were asymptomatic; therefore, careful observation and ECG monitoring are important to avoid oversight. POAF is strongly associated with perioperative complications; it may be the only marker for some of these complications. The clinical significance of POAF will be revealed on long-term surveillance.

## Supporting information

S1 Table Patient characteristics. (DOCX)

S2 Table Uni- and multivariate logistic regression analysis for an incidence of perioperative atrial fibrillation. (DOCX)
