# Supplementary material for: Incidence and complications of perioperative atrial fibrillation after non-cardiac surgery for malignancy
Source: PLoS One. 2019 May 7;14(5):e0216239. doi: 10.1371/journal.pone.0216239 (PMC6504100; doi:10.1371/journal.pone.0216239)
Supplement: S1 Table — (DOCX) [file pone.0216239.s001.docx]

**Supplemental Table 1. Patient characteristics**

|  | All (n = 813) | POAF (n = 87) | No POAF (n = 726) |
| --- | --- | --- | --- |
| Age, years | 68 ± 11 | 72 ± 7 | 67 ± 11 |
| Male, n (%) | 524 (62) | 60 (69) | 448 (62) |
| Body mass index, kg/m^2^ | 23 ± 3 | 23 ± 3 | 23 ± 3 |
| Systolic blood pressure, mmHg | 128 ± 17 | 129 ± 16 | 127 ± 18 |
| Diastolic blood pressure, mmHg | 77 ± 34 | 76 ± 13 | 77 ± 36 |
| Heart rate, bpm | 71 ± 12 | 73 ± 13 | 71 ± 12 |
| Past history of AF, n (%) | 14 (2) | 7 (8) | 7 (1) |
| Hypertension, n (%) | 369 (45) | 44 (51) | 325 (45) |
| Diabetes mellitus, n (%) | 153 (19) | 18 (21) | 135 (19) |
| Past history of heart failure, n (%) | 6 (1) | 0 (0) | 6 (1) |
| Past history of cardiogenic stroke, n (%) | 1 (0) | 1 (1) | 0 (0) |
| Coronary artery disease, n (%) | 44 (5) | 4 (5) | 40 (6) |
| Chronic obstructive pulmonary disease, n (%) | 212 (26) | 29 (33) | 183 (25) |
| CHADS2 score | 0 (1–2) | 0 (1–2) | 0 (1–2) |
| CHA2DS2-VASc score | 2 (1–3) | 2 (2–3) | 2 (1–3) |
| Brinkman index | 360 (0–890) | 540 (0–960) | 340 (0–870) |
| Alcohol consumption, g/day | 0 (0–20) | 0 (0–19) | 0 (0–20) |
| No malignancy, n (%) | 46 (6) | 2 (2) | 44 (6) |
| Malignancy |  |  |  |
| Stage 0, n (%) | 81 (10) | 3 (3) | 78 (11) |
| Stage 1, n (%) | 341 (42) | 43 (49) | 298 (41) |
| Stage 2, n (%) | 145 (18) | 20 (23) | 125 (17) |
| Stage 3, n (%) | 80 (10) | 9 (10) | 71 (10) |
| Stage 4, n (%) | 166 (20) | 12 (14) | 154 (21) |
| Head and neck cancer, n (%) | 35 (4) | 1 (1) | 34 (5) |
| Chest, n (%) | 523 (64) | 59 (68) | 464 (64) |
| Lung cancer, n (%) | 434 (53) | 55 (63) | 379 (52) |
| Metastatic lung cancer, n (%) | 64 (8) | 1 (1) | 63 (9) |
| Esophagus cancer, n (%) | 25 (3) | 3 (3) | 22 (3) |
| Abdomen, n (%) | 259 (32) | 27 (31) | 232 (32) |
| Pancreatic cancer, n (%) | 119 (15) | 8 (9) | 111 (15) |
| Carcinoma of the ampulla of Vater, n (%) | 9 (1) | 2 (2) | 7 (1) |
| Duodenal cancer, n (%) | 3 (0) | 0 (0) | 3 (0) |
| Intrahepatic cholangiocarcinoma, n (%) | 4 (0) | 1 (1) | 3 (0) |
| Cholangiocarcinoma, n (%) | 33 (4) | 4 (5) | 29 (4) |
| Gallbladder cancer, n (%) | 13 (2) | 2 (2) | 11 (2) |
| Hepatic cell carcinoma, n (%) | 34 (4) | 3 (3) | 31 (4) |
| Liver metastasis, n (%) | 45 (6) | 7 (8) | 38 (5) |
| Laboratory data |  |  |  |
| White blood cell count, 10^9^/L | 6.1 ± 2.0 | 6.3 ± 2.2 | 6.0 ± 2.0 |
| Hemoglobin, g/L | 132 ± 15 | 129 ± 14 | 133 ± 15 |
| Platelet count, 10^9^/L | 22.1 ± 6.8 | 22.0 ± 7.5 | 22.2 ± 6.8 |
| D-dimer, μg/L | 300 (200–600) | 400 (200–800) | 300 (200–600) |
| C-reactive protein, mg/L | 1.0 (0.0–2.0) | 1.0 (1.0–3.0) | 1.0 (0.0–2.0) |
| Sodium, mmol/L | 140 ± 5 | 140 ± 3 | 140 ± 6 |
| Pottasium, mmol/L | 4.3 ± 1.4 | 4.3 ± 0.4 | 4.4 ± 1.5 |
| Chloride, mmol/L | 105 ± 5 | 105 ± 3 | 105 ± 5 |
| Calcium, mmol/L | 2.35 ± 0.11 | 2.34 ± 0.11 | 2.35 ± 0.11 |
| Magnesium, mmol/L | 0.90 ± 0.14 | 0.89 ± 0.10 | 0.90 ± 0.14 |
| Albumin, g/L | 42 ± 5 | 41 ± 4 | 42 ± 5 |
| Creatinine, μmol/L | 63 (51–74) | 66 (54–82) | 62 (51–73) |
| B-type natriuretic peptide, ng/L | 22 (12–39) | 31 (17–45) | 21 (12–38) |
| Thyroid stimulating hormone, mIU/L | 1.7 (1.1–2.5) | 1.7 (1.0–2.4) | 1.7 (1.1–2.5) |
| Free T3, pmol/L | 3.8 ± 0.7 | 3.8 ± 0.7 | 3.8 ± 0.7 |
| Free T4, pmol/L | 15.4 ± 3.7 | 15.3 ± 2.6 | 15.4 ± 3.8 |
| Medication |  |  |  |
| Beta blocker, n (%) | 61 (8) | 9 (10) | 52 (7) |
| Beta stimulant, n (%) | 72 (9) | 12 (14) | 60 (8) |
| Renin angiotensine system inhibitors, n (%) | 205 (25) | 28 (32) | 177 (24) |
| Calcium channel blocker, n (%) | 258 (32) | 34 (39) | 224 (31) |
| Prior chemotherapy, n (%) | 96 (12) | 6 (7) | 90 (12) |
| Levothyroxine, n (%) | 20 (2) | 0 (0) | 20 (3) |
| Anticholinergic agents, n (%) | 56 (7) | 10 (11) | 46 (6) |

POAF, perioperative atrial fibrillation
