# Supplementary material for: Incidence and complications of perioperative atrial fibrillation after non-cardiac surgery for malignancy
Source: PLoS One. 2019 May 7;14(5):e0216239. doi: 10.1371/journal.pone.0216239 (PMC6504100; doi:10.1371/journal.pone.0216239)
Supplement: S2 Table — (DOCX) [file pone.0216239.s002.docx]

**Supplemental Table 2. Uni- and multivariate logistic regression analysis for an incidence of perioperative atrial fibrillation**

|  | Univariate logistic regression analysis | | Multivariate logistic regression analysis | |
| --- | --- | --- | --- | --- |
|  | OR (95% CI) | P value | OR (95% CI) | P value |
| Any 30-day complications | 2.69 (1.69–4.28) | < 0.001 | 2.60 (1.62–4.18) | < 0.001 |
| Chronic obstructive pulmonary disease | 1.48 (0.92–2.38) | 0.109 | 1.35 (0.82–2.20) | 0.238 |
| Hemoglobin (increase of 10 g/L) | 0.86 (0.74–0.99) | 0.035 | 0.89 (0.77–1.04) | 0.139 |
| Creatinine (increase of 20μmol/L) | 1.07 (1.01–1.13) | 0.018 | 1.06 (1.00–1.12) | 0.052 |
| CHA2DS2-VASc score (increase of 1 unit) | 1.21 (1.01–1.44) | 0.036 | 1.15 (0.96–1.39) | 0.132 |

Multivariate logistic regression analysis was adjusted for any 30-day complications, chronic obstructive pulmonary disease, hemoglobin,　creatinine, and CHA2DS2-VASc score.

P < 0.05 was considered statistically significant.

CI, confidence interval; OR, odds ratio; POAF, perioperative atrial fibrillation
